# Supplementary material for: Rational Design of Alginate Lyase from Microbulbifer sp. Q7 to Improve Thermal Stability
Source: Mar Drugs. 2019 Jun 25;17(6):378. doi: 10.3390/md17060378 (PMC6627800; doi:10.3390/md17060378)
Supplement: Supplementary file 1 [file marinedrugs-17-00378-s001.pdf]

Table S1 The results of predication of disulfide bond

Predicted Disulfide Bond	Original Residues	Score	Energy	Bad Contacts	Thermal Mobility	Sequence Separation	Residue Depth	Volume Change	Environment	Chi3
CYS179-CYS304	VAL179, VAL304	91.98	4.01	0	1.7	125	9.01	-70.5	Sheet-Sheet	127.4
CYS166-CYS305	HIS166, GLN305	91.57	4.22	0	1.63	139	4.98	-102.3	Sheet-Sheet	144
CYS253-CYS274	ASN253, GLY274	91.47	4.27	0	1.63	21	2.95	16.9	Turn-Turn	-100.3
CYS102-CYS300	ASP102, ALA300	90.61	4.7	0	1.47	198	3.08	-0.5	Coil-Turn	90.4
CYS121-CYS152	LEU121, GLY152	90.53	4.74	0	1.35	31	3.64	-21.2	Coil-Turn	-84.2
CYS103-CYS113	GLY103, THR113	90.2	4.9	0	1.48	10	5.65	22.5	Coil-Coil	118.3
CYS142-CYS279	SER142, ASP279	89.88	5.06	0	1.54	137	3.64	-5.4	Coil-Turn	-95.3
CYS59-CYS86	VAL59, TYR86	88.42	5.79	0	1.47	27	6.46	-128.6	Coil-Turn	-124.1
CYS46-CYS95	PRO46, MET95	87.37	6.32	0	1.6	49	6.3	-82.8	Coil-Sheet	-111.9
CYS207-CYS224	VAL207, ILE224	87.34	6.33	0	1.76	17	5.74	-95.5	Sheet-Sheet	150.7
CYS141-CYS155	GLY141, ASP155	87.3	6.35	0	1.6	14	3.19	25.2	Coil-Turn	-150.4
CYS97-CYS115	PHE97, THR115	86.95	6.52	0	1.67	18	8.57	-106.2	Sheet-Sheet	91.6
CYS161-CYS309	THR161, TYR309	86.73	6.63	0	1.75	148	3.77	-110.2	Sheet-Sheet	-159.1
CYS173-CYS229	SER173, SER229	86.55	6.72	0	1.41	56	3.46	17.5	Coil-Turn	-142
CYS119-CYS286	GLU119, TYR286	85.48	7.26	0	1.43	167	8.23	-131.6	Sheet-Sheet	-153.1
CYS128-CYS135	ILE128, GLY135	85.24	7.38	0	1.3	7	3.3	-20.8	Coil-Turn	150.2
CYS182-CYS194	GLY182, LEU194	84.82	7.59	0	1.83	12	10.89	-21.2	Sheet-Sheet	148.2
CYS55-CYS117	TRP55, LEU117	84.72	7.64	0	1.55	62	7.73	-187.5	Sheet-Sheet	140.9
CYS122-CYS136	ARG122, ASN136	82.99	8.51	0	1.26	14	4.8	-114	Turn-Turn	-108.7
CYS139-	VAL139,	82.52	8.74	0	1.51	12	6.02	-21.5	Coil-Sheet	-78.4

CYS151	ALA151									
CYS184-CYS191	ILE184, PRO191	81.89	9.05	0	1.59	7	9.34	-80	Coil-Sheet	178.4
CYS62-CYS104	ASP62, TYR104	81.76	9.12	0	1.3	42	3.93	-107.5	Coil-Coil	-51.5
CYS247-CYS258	GLU247, THR258	81.48	9.26	0	1.97	11	5.48	-55.2	Sheet-Sheet	149.8
CYS171-CYS176	GLY171, VAL176	68.36	5.82	1	1.62	5	4.27	4.1	Coil-Turn	166.9
CYS47-CYS52	SER47, A:LEU52	67.56	6.22	1	1.4	5	4.98	-51.9	Coil-Turn	113.6
CYS214-CYS277	ASN214, GLY277	62.96	8.52	1	1.46	63	3.06	16.9	Coil-Turn	-136.3

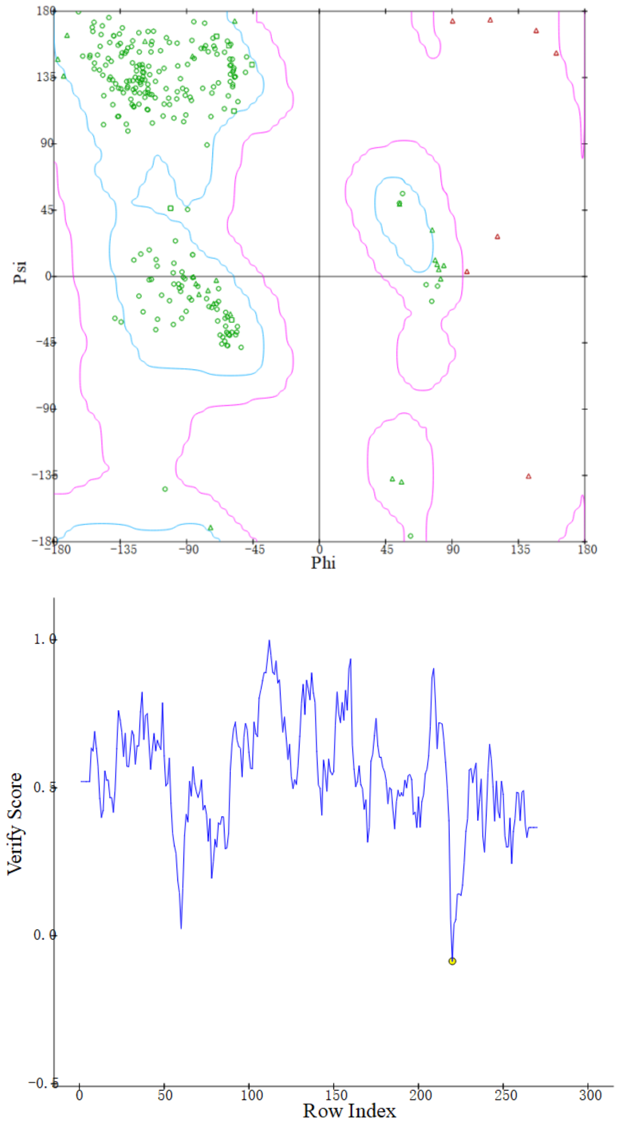

Figure S1 The Ramachandran plot (A) and Profile-3D (B) of cAlyM 3D structure.

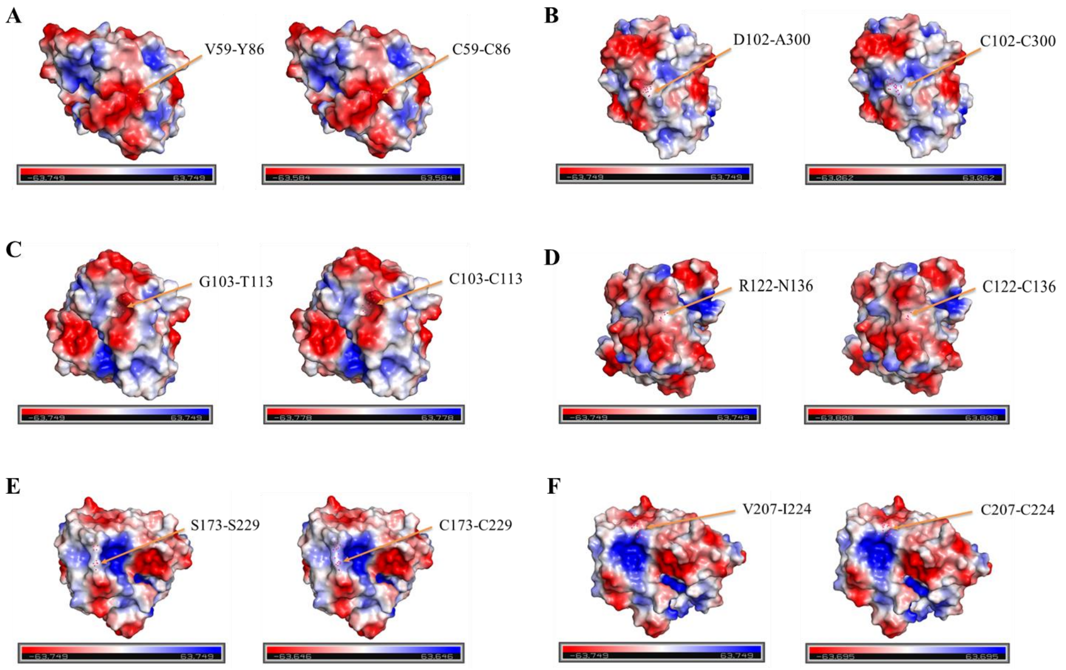

Figure S2 Comparison of surface charge around the mutant sites of cAlyM and its mutants. A: V59C-Y86C; B: D102C-A300C; C: G103C-T113C; D: R122C-N136C; E: S173C-S229C; F: V207C-I224C.
